# A new method for identifying industrial clustering using the standard deviational ellipse

**DOI:** 10.1038/s41598-023-27655-8

**Published:** 2023-01-11

**Authors:** Ziwei Zhao, Zuoquan Zhao, Pei Zhang

**Affiliations:** 1 School of Public Policy and Management, University of Chinese Academy of Sciences, Beijing, 100049 China; 2 Institutes of Science and Development, Chinese Academy of Sciences, Beijing, 100190 China; 3 Key Laboratory of Regional Sustainable Development Modeling, Institute of Geographic Sciences and Natural Resources Research, China Academy of Sciences, Beijing, 100101 China

**Keywords:** Socioeconomic scenarios, Information technology

## Abstract

Industrial agglomeration has attracted extensive attention from economists and geographers, yet it is still a challenge to identify the multi-agglomeration spatial structure and degree of industrial agglomeration in continuous space—there is still a lack of a more targeted industrial clustering method. The clustering method and the standard deviational ellipse (simply, ellipse) model have advantages in identifying the spatial structure and representing spatial information respectively. On this basis, we propose an ellipse-based approach to identifying industrial clusters. Our ellipse-based approach rests upon group nearest neighbor using the group-based nearest neighbor (GNN) ordering and spatial compactness matrix, where a number of point sequences with varying lengths, generated under the GNN ordering, are characterized by an ellipse and the elliptical parameters of these point sequences formulate the values and structure of the compactness matrix. Clustering is reformulated to identify ellipses with a specified parameter among a number of potential candidate ellipses, with significant changes (especially in the area) used as the cutoff criterion for determining the clusters’ border point. Our approach is illustrated in the location pattern of firms in Shanghai City, China in comparison with four well-known clustering methods. With the combination of elliptical parameters and spatial compactness, our approach may bring a new analytical ground for future industrial clustering research.

## Introduction

The phenomenon of industrial agglomeration on the earth arouses research in various fields of science^1–4^. In the field of human geography, industrial agglomeration is the most significant feature of world economic activities. British economist Marshall first paid attention to the phenomenon of industrial agglomeration and the concept of ‘industrial cluster’ was first proposed by Porter in 1990, which triggered increasing attention to industrial agglomeration^5,6^. In recent decades, a large number of empirical studies on industrial agglomeration have been published. These studies revolve around whether industries and their firms are agglomerated (or clustered) in regions or spaces, and the extent and scale of agglomeration from an overall perspective. The kernel function can effectively identify the location and quantity of agglomeration^7^, but due to the arbitrary bandwidth, the boundary of agglomerations cannot be determined. The spatial Gini coefficient^8^, the industry concentration measure^9^, the Herfindahl index^10,11^, the location entropy^12^ and the work of Ellison and Glaeser^13^ can measure the degree of agglomeration, but there is the modifiable areal unit problem (MAUP): the shape, size, and position of the selected regions will have a greater impact on the measurement^14,15^. The works of Ripley, Marcon and Puech and Duranton and Overman can test the existence of industrial agglomeration in continuous space to overcome the MAUP^16–18^, but cannot identify the actual spatial structure of the multi-agglomeration pattern. So far, there has been little discussion about the method simultaneously fulfilling three requirements: (i) measuring the degree of industrial agglomeration (ii) identifying the spatial structure of agglomeration multi-agglomeration pattern, i.e. the number, location, and spatial extent of agglomeration^19^ (iii) data analysis work is done in continuous space to avoid boundary deviations and arbitrary spatial units.

As an important method for spatial data analysis, spatial clustering has potential in agglomeration analysis. As Delgado pointed out^20^, the economies of agglomeration manifest more clusters—in geographic concentrations of related industries and associated institutions. Naturally, we try to apply the clustering method used for spatial point analysis to identify industrial clusters. A few studies have applied clustering algorithms to industrial agglomeration analysis^21^. For example, Karaca and Zeynep^21^ used the K-means clustering method to determine how the wood products manufacturing sector was clustered in the Nace Rev region of Turkey and ultimately identified three clusters. However, such studies are still rare and imperfect. Firstly, most of the existing studies use a single clustering method. Due to the lack of evaluation of clustering results and comparison of clustering results of different methods, these studies cannot verify the effectiveness of a single method in industrial clustering. Secondly, the existing research applies the classical clustering method to the analysis of industrial data rigidly, without adaptive improvement. Due to the randomness and irregularity of industrial data, and there is no prior information such as the number of clusters to be used, industrial clustering methods that are more targeted than existing spatial clustering methods are needed. Notably, the underlying rich spatial information within industrial agglomerations relies on complete spatial representation, while most clustering methods attempt to use partial information to determine a complete structure of data. The information includes distance, centroid, density, shape, and direction of local clusters. For example, K-means^22–24^ uses distance and centroid metrics to identify local clusters; DBSCAN^25^ uses a density metric; the complete linkage^26^ uses a distance metric; multilayer network^27^ uses a shape metric; both the nearest neighbor and nearest centroid neighbor^28,29^ use a direction metric. Recently, some clustering methods use relatively integrated information. A typical example is Rodriguez and Laio^30^ who attempt to combine the centroid, distance, and density metrics into a more integrated clustering framework. Practically, some metrics, such as shape^31^ and direction^28,29^, have been frequently ignored or not been well represented in cluster analysis. This loss of information may be related to the failure of many clustering methods to deal with industrial data with a lower clustering tendency. Against this background, the elliptic model shows a great advantage in representing a neighborhood and a cluster in spatial cluster analysis. The power of ellipses in spatial data representation is evidenced by the popularity of ellipses in spatial data analysis and the elliptical mixture models in cluster analysis^32–35^. Therefore, it would be desirable to have a consistent framework of clustering utilizing the elliptic model.

For the lack of a satisfactory cluster representation and a more targeted industrial clustering method, this study aims to propose an ellipse-based clustering (EBC) which (i) measures the degree of industrial clustering; (ii) effectively identifies the multi-cluster pattern, i.e. the number, location, and spatial extent of agglomeration; (iii) could be operated in continuous space. Our approach directly uses ellipses to represent, quantify, and identify the local neighborhood as well as clusters of point data. The ellipse-based approach operates on spatial compactness ordering, from which all data points are turned into a number of point sequences by using a group-based nearest neighbor, and each group of points in the sequences, examined as a potential cluster, is characterized intuitively by an ellipse in terms of elliptic parameters, including its center, area, density, shape, and orientation. Clustering is reformulated as the problem of identifying high-density ellipses among a number of potential ellipses, with significant changes (especially in the area) used as the cutoff criterion for determining the clusters’ border point. Notably, our approach allows for the number of clusters arising naturally, and only one parameter (the cutoff value) to be specified statistically during the clustering process.

It is noted that our ellipse-based approach differs from the mixture model-based clustering approach^36,37^ and the elliptic spatial scan statistic^38^, the two widely used ellipse-related clustering methods in several important ways. First, our approach is fully data-driven in the sense that all ellipses are computed from the point groups resulting from spatial compactness ordering. Unlike ours, the model-based clustering methods need to estimate the clustering structure of data with the assumption that different clusters result from different probability models^34^. While the elliptic scan statistic tends to employ an ellipse-shaped scanning window for clustering, in which these ellipses have nothing to do with the underlying clustering structure^38^. In contrast to elliptic scan statistics, which use predefined ellipses with different parameters to randomly search and match the local neighborhood of data^38^, our approach operates orderly on the spatial compactness matrix, from which the ellipses in use capture the spatial structure of point groups in the matrix’s rows.

In cluster representation, the elliptic model shows a great advantage in representing a neighborhood and a cluster in spatial cluster analysis. The use of ellipses in spatial data representation is popular in spatial data analysis and the elliptical mixture models in cluster analysis^32–35^. Ellipses differ from other cluster representations such as a graph^39,40^, a tree^41^, a path^42,43^ or a line segment^44^ in that ellipses quantify directly the spatial structure of a neighborhood using elliptic parameters, while such representation as a graph, for instance, can capture only some information of a cluster, e.g., minimum spanning tree, with some other metrics, e.g., nearest neighborhood distance^40,45^ indispensable in cluster analysis. Therefore, many clustering methods tend to use individual location-based metrics in less integrated ways. For representing the range of a neighborhood, we choose the ellipse’s area, which can vary with direction, different from such range metrics as $\varepsilon$-neighborhood’s radius^25^, density reachable distance^46^, gradient distance, and Gaussian density distance^47^, neighborhood width^48^ and so on; For representing the orientation of a neighborhood, we use the direction of the major axis of the ellipse, which has not been examined in cluster analysis^23,24^; For representing the shape of a neighborhood, we use the ratio of the length of the minor axis to that of the major axis, in sharp contrast with the shape metrics of density core^31^ and inter-connection coefficient^47^. For representing the center of a neighborhood, we choose the average center because the average center is the most widespread cluster representation in data clustering^23,24^.

In this paper, ellipse-based clustering is employed to identify the industrial agglomeration of manufacturing firms in Shanghai City. Shanghai City is located on the edge of the Yangtze River Delta, the largest urban agglomeration in the eastern coastal areas of China, where manufacturing plays an important role in economic development. Our approach is compared with K-means clustering with geographical coordinates as variables (K-means), Ester’s density-based spatial clustering of application with noise (DBSCAN)^25^, King’s complete-link clustering^26^ (Complete-link), and the expectation-maximization algorithm based on the Gaussian mixture model (GMM-EM)^37^. These clustering methods are respectively the leading algorithms in partitional clustering, density-based clustering, hierarchical clustering, and ellipse-related clustering. On the one hand, we use Davies-Bouldin index (DBI) to evaluate and compare the results of different clustering methods; On the other hand, we compare the results of different methods with kernel density analysis to judge the effectiveness of different methods in identifying industrial clusters.

## The model

### Ordering and matrices

We define a group-based version of nearest neighbor as follows:

#### **Definition 1**

(*Group-based nearest neighbor*) Let *E*, *D* be sets, $E\cap D=\varnothing$, $D=\{\alpha _1,\alpha _2, \ldots ,\alpha _n\}$, where $\alpha _i\in {\mathbb {R}}^2$, $i=1,2, \ldots ,n$. For $\forall \beta \in E$, if  $\exists \hat{\beta }\in E$, s.t. $d(D,\hat{\beta }) \le d(D,\beta )$ then $\hat{\beta }$ is the group-based nearest neighbor (G-NN) of *D* within *E*, $\hat{\beta }$ = *GNN*(*D*, *E*).

Briefly, for two disjoint sets, the group-based nearest neighbor of a set within another set is the closest point in the later set to the former. As for the measurement of distance, we define the distance between a data point and a set:

1 $$\begin{aligned} d_{avel}(D,\beta )=\sum \limits _{i=1}^{i=n} \frac{d(\alpha _i,\beta )}{n}. \end{aligned}$$

  The distance between $\alpha _i$ and $\beta$ can be measured by Euclidean distance, Minkowski distance and Manhattan distance, etc. Here $d_{avel}$ is used to calculate the group-based nearest neighbors, with Euclidean distance calculating $d(\alpha _i,\beta )$. Based on the group-based nearest neighbors, we construct a compact ordering of points and present the ordering in the form of the spatial compactness matrix.

#### **Definition 2**

(*Spatial compactness matrix*) Let $A_{ij}$,$B_{ij}$, X be sets of spatial points, X = $\{x_1,x_2,\ldots ,x_n\}$. For $i=1,2,\ldots ,n$, $j=1,2,\ldots ,n$, $x_i\in {\mathbb {R}}^2$, $z_{i1}=x_i$, $A_{ij}=\{z_{i1},z_{i2},\ldots ,z_{ij}\}$, $B_{ij}=X\setminus A_{ij}$. $\hat{\beta }$ is the group-based nearest neighbor of $A_{ij}$ within $B_{ij}$:

2 $$\begin{aligned} A_{i,j+1}=\{z_{i1},z_{i2},\ldots ,z_{ij}, \hat{\beta }\}=A_{ij}\cup \{z_{i,j+1}\} \end{aligned}$$

$A_{ij}$ denotes a compactness sequence of *j* points starting from $x_i$. $Z=(z_{ij})_{nn}$ denotes a spatial compactness matrix (SCM).

Especially, For spatial points defined on the sphere $S^2 (R)$ with radius *R*, we use the length of arc to measure the distance between any two spatial points. Let ($a_1,b_1$), ($a_2,b_2$) be the latitude and longitude coordinates of $z_{ik}$ and $\beta$.

3 $$\begin{aligned} d(z_{ik},\beta )=R\cdot arccos(cosb_1cosb_2cos(a_1-a_2)+sinb_1sinb_2), \end{aligned}$$

where *R* is the radius of the sphere.

The spatial compactness matrix leads to data compression and simplified operation: (i) instead of identifying each data point, we identify the compactness sequences of points for by using standard deviational ellipses, ending up with an ordered and comprehensive representation of clusters; (ii) the number of non-repetitive clusters composed of *n* points is *m*, $m=C_n^1+C_n^2+\cdots +C_n^n=2^n-1,$ while the number of sequences from SCM is $n^2$. Sorting by compactness eliminates those clusters that are not compact enough, reducing the number of candidate clusters from *m* to $n^2$.

### Elliptic parametrization

With the assistance of ellipses, we parameterize the compactness sequences. Consider a $n\times n$ SCM $Z=(z_{ij})_{nn}$. $A_{ij}=\{z_{i1},z_{i2},\ldots ,z_{ij}\}$ is a corresponding cluster of a standard deviational ellipse. For potential clusters, we calculate the rotation angle $\theta$ and the standard deviation of $x{\text{-}}axis$ and $y{\text{-}}axis$ ($\sigma _x$ and $\sigma _y$) of their ellipses. Feature $z_{ik}$ in $A_{ij}$ has an ordinate of $\hat{y_k}$ and a horizontal coordinate of $\hat{x_k}$, $k=1,2,\ldots ,n$, ${\bar{X},\bar{Y}}$represent the average center of the elements in $A_{ij}$, $\tilde{y}_k=\hat{y_k}-\bar{Y}$,$\tilde{x}_k=\hat{x_k}-\bar{X}$,then:

4 $$\begin{aligned} A= \left(\sum \limits _{k=1}^{j}{\tilde{x}_k}^2-\sum \limits _{k=1}^{j}{\tilde{y}_k}^2 \right),\ C=2\sum \limits _{k=1}^{j}\tilde{x}_k\tilde{y}_k,\ B=\sqrt{A^2+4C^2},\ tan\theta =\frac{A+B}{C}, \end{aligned}$$

5 $$\begin{aligned}{} & {} \sigma _x=\sqrt{\frac{2\sum _{k=1}^{j}(\tilde{x}_{k}cos\theta -\tilde{y}_{k}sin\theta )^2}{n}},\ \sigma _y=\sqrt{\frac{2\sum _{k=1}^{j}(\tilde{x}_{k}sin\theta +\tilde{y}_{k}cos\theta )^2}{n}}. \end{aligned}$$

  On the basis of representation and related research on ellipse model^32–34,36,38^, we use the center, area, density, the direction of the major axis, the ratio of the length of the minor axis to that of the major axis to establish parametric matrices.

Let $n\times n$ center matrix $M_{mc}=(m_{ij}^1)_{nn}$, area matrix $M_{area}=(m_{ij}^2)_{nn}$, density matrix $M_d=(m_{ij}^3)_{nn}$, shape matrix $M_s=(m_{ij}^4)_{nn}$, azimuth matrix $M_a=(m_{ij}^5)_{nn}$. $m_{ij}^1=\{\bar{X},\bar{Y}\}$, $m_{ij}^2=\pi \cdot \sigma _x\cdot \sigma _y$, $m_{ij}^3=j/m_{ij}^2$, $m_{ij}^4=\sigma _x/\cdot \sigma _y$, $m_{ij}^5=\theta$. Notably, for spatial points defined on the sphere $S^2 (R)$ with radius *R*, we use the elliptic area under the equal area projection of the spherical surface to approximate the expression $m_{ij}^2$.

### Determining candidate clusters

Rodriguez and Laio^30^ propose an approach based on the idea that cluster centers are characterized by a higher density than their neighbors and by a relatively large distance from points with higher densities. Here, clustering starts with the local maximum density in each sequence. Significant changes in the area are used as the cutoff criterion for determining the clusters’ border points. To measure the changes, we establish the area gradient matrix and define $\xi$-steep points.

#### **Definition 3**

(*Area gradient matrix*) According to $n\times n$ area matrix $M_{area}=(m_{ij}^2)_{nn}$, establish $n\times (n-2)$ area gradient matrix $Area=(a_{ij}^3)_{n\times (n-2)}$,

6 $$\begin{aligned}{} & {} a_{ij}^3=\frac{(a_{ij}^2-a_{i}^2)^2}{\sum (a_{ij}^2-a_{i}^2)^2} \end{aligned}$$

where $$a_{i}^2={\sum {a_{ij}^2}}/n$$, $$a_{ij}^2=a_{i,j+1}^1-a_{ij}^1$$, $$a_{ij}^1=m_{i,j+1}^2-m_{ij}^2$$, $$i=1,2,\ldots ,n , \;\;\;j=1,2,\ldots ,n-1$$.

#### **Definition 4**

(*Steep points*) For $z_{a,b+2}$ within $n\times n$ *SCM* $Z=(z_{ij})_{nn}$, if the corresponding $a^3_{a,b}>\xi$, then $z_{a,b+2}$ is defined as a $\xi$-steep point.

Among the sequences sharing a common starting point $A_{ij}, j=1,2,\ldots ,n$, the sequence with the highest density, e.g., $A_{i,m^i}$, is chosen as the initial cluster, $m_{i,m^i}^3\ge m_{ij}^3$. For $j=m^i+1$ , determine whether $a^3_{i,j}<\xi$ holds. If so, continue to determine $a^3_{i,j}<\xi$ where $j=m^i+2$. The process is repeated. Let $H_1$ be the set of $(i,j)$ which cannot make the discriminant true, $H_1=\{(i,j)|\ a^3_{i,k}<\xi ,\ k=m^i+1,m^i+2,\ldots ,j,\ a^3_{i,j+1}\ge \xi \},\ card(H_1)=n_1$. Excluding the sequences with a density lower than the density from $n_1$ sequences, we get the set $H_2$ and $n_2$ candidate clusters, $H_2=\{(i,j)|\ (i,j)\in H_1,\ m_{ij}^3\ge {\bar{m}}^3 \}$, $card(H_2)=n_2$. Let F be the set of candidate clusters $F=\{A_{ij}|\ (i,j)\in H_2\}$. It is worth noting that sequences with the same elements but different orderings are considered to be the same cluster. In this section, candidate clusters with better performance in density and continuity stand out from compact sequences.

### Optimization

We optimize the clustering results on the basis of candidate clusters and the measure of similarity.

#### **Definition 5**

(*Clustering adjacency matrix*) For clusters $C_{i},\,x_i=0,1, \quad i=1,2,\ldots \,n$, $\sum {x_i}=m$, a clustering adjacency matrix H is defined:

7 $$\begin{aligned} H(x_1C_1,x_2C_2, \ldots ,x_{n}C_{n})=(h_{ij})_{mm}, \ h_{ij}= {\left\{ \begin{array}{ll} 0,x_ix_j=1,C_i\cap {C_j}=\varnothing \\ 1,x_ix_j=1,C_i\cap {C_j}\ne \varnothing \end{array}\right. } \end{aligned}$$

We traverse all combinations of non-overlapping candidate clusters. Among these combinations, we select the one with the objective function maximum. Here, we use the sum of the areas of the ellipses corresponding to the clusters as the objective function:

8 $$\begin{aligned}{} & {} max\ f=\sum \limits _{i=1}^{n_2}x_i\cdot m_i^* \end{aligned}$$

9 $$\begin{aligned}{} & {} {\left\{ \begin{array}{ll} H(x_1C_1,x_2C_2, \ldots ,x_{n_2}C_{n_2})=I \\ x_i = 0,1\quad i=1,2, \ldots ,n_2 \end{array}\right. } \end{aligned}$$

  According to the solution to this optimization problem, if $x_i=1$, then $C_i$ is the class we need. It is worth noting that $\xi$ has a vital influence on the generation of candidate clusters. Actually, the selection of parameters is not arbitrary, but determined by the characteristics of data sets. Here we use the average of density and sum of area to measure the similarity and determine the optimal $\xi$.

## Results

### Data and baselines

We identify industrial clusters for Shanghai manufacturing listed firms. As of January 8, 2019, there were a total of 479 manufacturing firms in Shanghai City that were listed on the Shanghai and Shenzhen stock markets or the ChiNext stock market (without distinguishing between A-shares and B-shares), corresponding to 474 spatial points, with five locations each occupied by two firms. The securities codes and addresses of the listed manufacturing firms in Shanghai City were provided by the WIND database. We geocode the addresses with Gaode Map API to obtain a series of latitude and longitude coordinates based on the WGS-84 coordinate system and the Albers projected coordinate system. For ease of illustration, we use the stock code of a listed company to represent each company. Notably, our study did not consider the only firm in the Chongming district, which is an obvious noise point.

During the ellipse-based clustering, the average density and the sum of area are used to measure the similarity of clusters. The higher the average density, the better the compactness of the clustering pattern. Figure 3e,f shows the average density and the sum of area calculated by selecting clustering under different parameters ($\xi$).

Before clustering, we use the Hopkins statistic to access clustering tendency. The Hopkins Statistic is a spatial statistic that tests the spatial randomness of a variable as distributed in space. We conduct 100 Hopkins Statistic tests, using 0.3 as the sampling ratio. The average of the Hopkins statistic is 0.79 (> 0.75), indicating that there is a clustering tendency in the data set at a 90% confidence level.

### Identification of industrial clusters using EBC

**Figure 1:**
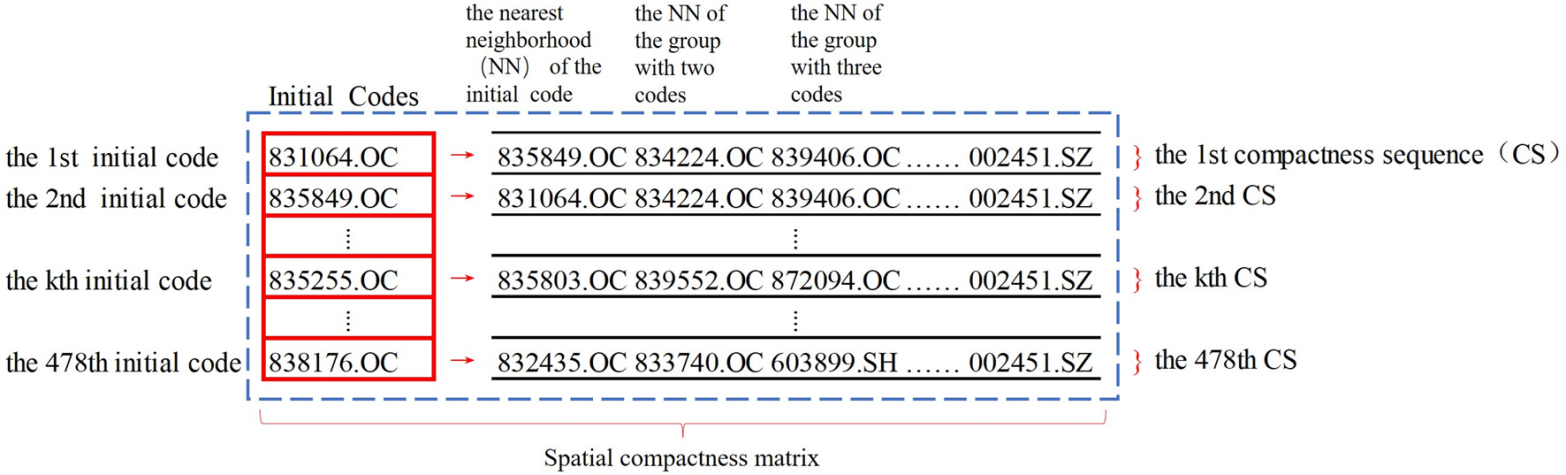
The SCM of listed firms in Shanghai.

In the first step of the EBC algorithm, we compute the spatial compactness matrix. The process is illustrated in Fig. 1. Each of the 478 manufacturing firms in Shanghai is the first column of each row of the matrix, corresponding to the starting point of each compactness sequence. For every compactness sequence, the nearest neighbor of the group with first j points is selected as the next point. Add this point to the original compactness sequence and update the compactness sequence. Repeat the process to update the length of each compactness sequence to 478, and the compactness matrix can be obtained. we calculate the index matrix.

Based on the SCM and its corresponding elliptic parameter index matrix, we select and intercept the compactness sequence satisfying continuity and density. It’s worth noting that termination of expansion depends on parameters related to area mutation. One possible approach is to use the probability density function to select from all the rates of change of area corresponding to the matrix. In this case, we select parameters ranging from 0.01 for 95$\%$ to 0.025 for 99$\%$ with 0.001 intervals.

**Figure 2:**
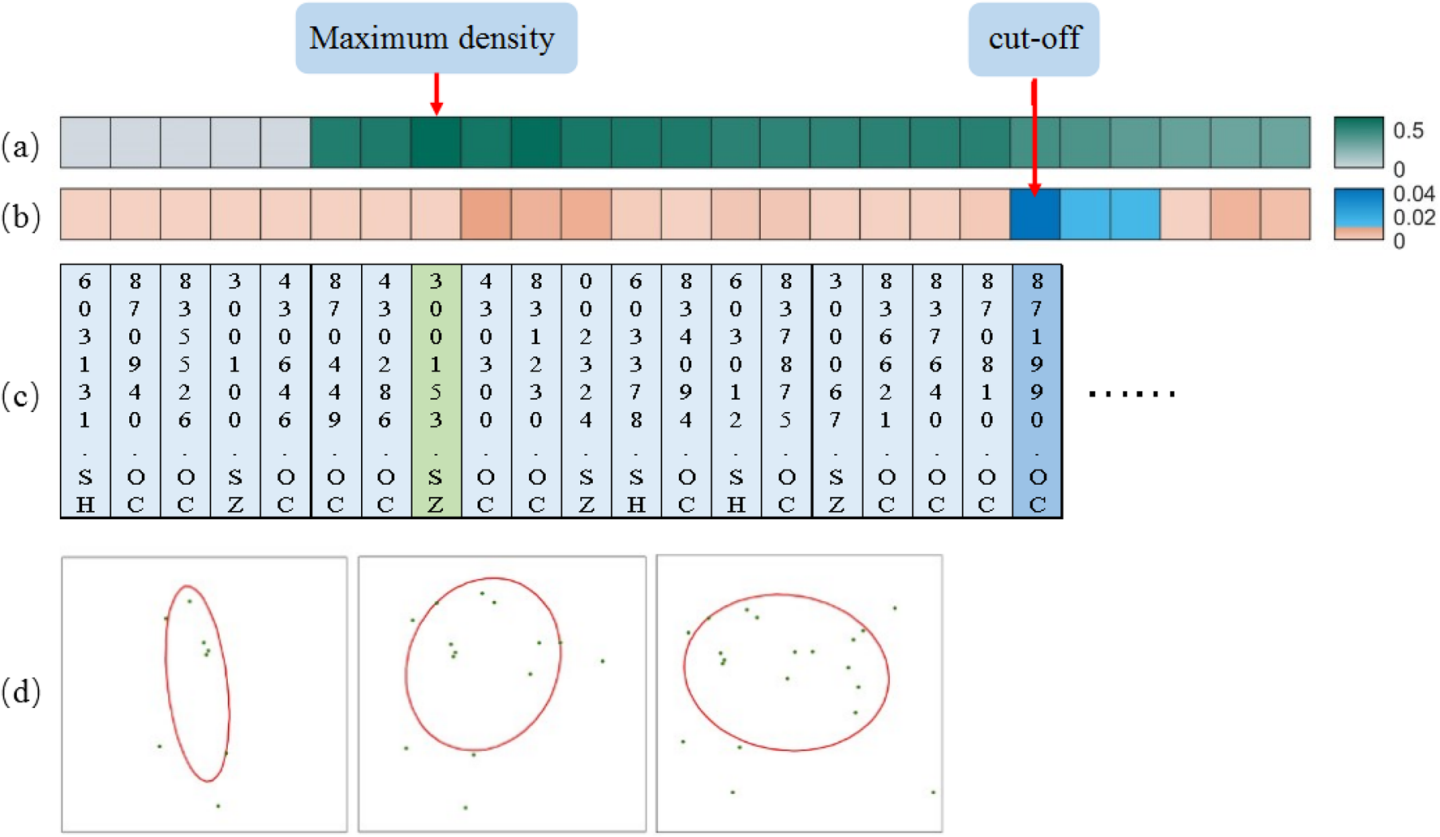
Initialization and termination of a sequence: initialize by elliptical density and select candidate clusters by mutation of elliptical area.

Figure 2 shows the process of selecting candidate clusters from all sequences starting with 603131.SH, taking location distribution of firms in Shanghai City as an example. The first heat map, the map of density, shows that the densest location in the row is chosen as the beginning. The second heat map, the heat map of area gradient, shows that each sequence stops expanding when the area mutation is greater than $\xi$. Besides, Fig. 2 displays the initialization and termination. Figure 2c shows the securities codes for the firms added, and Fig. 2d shows the change of the standard deviation ellipse corresponding to the sequence. With the increase of points, the ellipse gradually changes its shape and area. Candidate clusters with better performance in density and continuity stand out from compact sequences.

**Figure 3:**
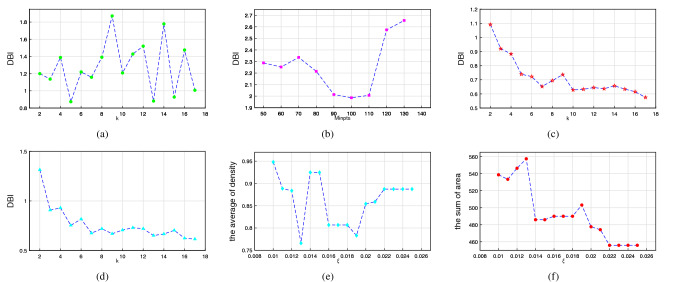
Similarity measure under different parameters: (**a**) optimal k through DBI (GMM-EMM1), (**b**) optimal MinPts through DBI (DBSCANM2, $\varepsilon$ = 0.015), (**c**) optimal k through DBI (complete-linkM3), (**d**) optimal k through DBI (K-meansM4), (**e**) optimal $\xi$ through the average of density (EBC), (**f**) optimal $\xi$ through the sum of area (EBC).

After all candidate sequences are determined, the final clustering pattern is output according to the principle of non-overlap and maximum sum of areas. The higher the average density, the better the compactness of the clustering pattern. The sum of average area spatial dispersion. Figure 3e,f shows the sum of average density and area calculated by selecting clustering patterns with different area variation parameters ($\xi$). Based on the comprehensive judgment of the results, we set $\xi$ = 0.01, which is more efficient. EBC identifies 13 clusters and 139 noise points (accounted for 29.1$\%$). The attributes of these 13 clusters are shown in Table 1. The sum of the areas of the corresponding ellipses in the 13 clusters is 538.64 km^2^, accounting for 32$\%$ of the total pattern ellipse area (1680.68 km^2^); the average density of all clusters is 0.6293, which is 2.213 times the average density of the total pattern (0.2838). The densities of the clusters range from 1.30 to 10.00 while the areas range from 7.27 to 299.69 km^2^. The average of shapes is 0.46 while the minimum is 0.1.

**Table 1:** Profile (variance) of clusters.

Cluster	X (°)	Y (°)	Shape index	Azimuth (°)	Size	Density index (/km^2^)	Area index (km^2^)
1	121.36	31.14	0.51	17.68	166	0.55	299.69
2	121.35	30.81	0.36	− 17.79	22	0.43	50.64
3	121.13	31.18	0.77	− 82.05	20	0.44	45.53
4	121.51	31.31	0.69	− 13.15	20	0.68	29.40
5	121.32	31.32	0.44	− 55.41	16	0.75	21.48
6	121.62	31.26	0.48	53.15	12	0.60	20.00
7	121.19	31.43	0.54	− 17.85	8	0.54	14.82
8	121.44	30.95	0.32	35.22	14	1.14	12.25
9	121.62	31.12	0.21	− 15.49	11	0.90	12.16
10	121.70	31.21	0.10	− 13.45	8	0.83	9.67
11	121.59	31.20	0.75	− 67.78	17	2.09	8.12
12	121.50	31.24	0.44	− 62.21	12	1.58	7.61
13	121.52	31.09	0.44	24.74	13	1.79	7.27

## Discussion

In this section, we will compare the results given by the proposed EBC algorithm with those produced by: (i) the Expectation-Maximization algorithm based on Gaussian mixture models^37^; (ii) Ester’s DBSCAN^25^; (iii) King’s complete-link clustering^26^; (iv) K-means clustering with geographical coordinates as variables. An ideal clustering method should ensure that the internal elements are compact and the different clusters are separated, while the ideal clustering method for industrial data should identify high-density and compact regions, and the points in the regions that are not dense enough are regarded as the noise. Therefore, we use the Davies-Bouldin index to evaluate the results of different clustering methods and compare the results with kernel density analysis to further demonstrate the performance of different methods in industrial agglomeration analysis.

Considering that the prior categories of the samples in the Shanghai manufacturing listed firms dataset are not available, and we are more concerned with comparing the results obtained by different methods, here we use the Davies-Bouldin index, a widely used validity measure^49,50^, to test the clustering quality of different methods on firms’ location data set^51^. The main idea of the Davies-Bouldin index is that a reasonable clustering result should be uniform and compact inside, and there should be a good separation between clusters. Given the internal dispersion $s_i$ of a cluster, the separation $d_{ij}$ between two clusters, the similarity $R_{ij}$ between clusters is generally defined as:

10 $$\begin{aligned}{} & {} R_{ij}=\frac{s_i+s_j}{d_{ij}}, \quad i,j=1,2, \ldots ,n,\ i\ne j , \end{aligned}$$

11 $$\begin{aligned}{} & {} s_i=\frac{\sum _{x\in {C_i}}d(x,v_i)}{N_i},\quad i=1,2, \ldots ,n, \end{aligned}$$

where $v_i$ represents the centroid of clusters $C_i$ ; $N_i$ represents the number of entities in cluster $C_i$. The DBI can be defined as:

12 $$\begin{aligned}{} & {} DBI=\frac{\sum _{i=1}^{N_c}R_i}{N_c}, \end{aligned}$$

13 $$\begin{aligned} R_i=max(R_{ij}), \quad j=1,2, \ldots ,N_c, \end{aligned}$$

where $N_c$ represents the number of clusters. The smaller the value of DBI, the better the clustering result.

When clustering industrial data sets, due to the lack of prior information such as the number of clusters, we test the effectiveness of each clustering method under different parameters. As shown in Fig. 3a–d, we select appropriate parameters for four clustering methods. When k = 4, GMM-EM performs best, DBI = 0.873; When MinPts = 100, $\varepsilon$ = 0.015, DBSCAN performs best, DBI = 1.99; When k = 16, complete-link performs best, DBI = 0.575; When k = 16, K-means performs best, DBI = 0.8730.615; In comparison, when $\xi$ = 0.01, EBC performs best, DBI = 0.477. The results show that on the industrial data set, EBC is superior to the other four algorithms under DBI.

**Figure 4:**
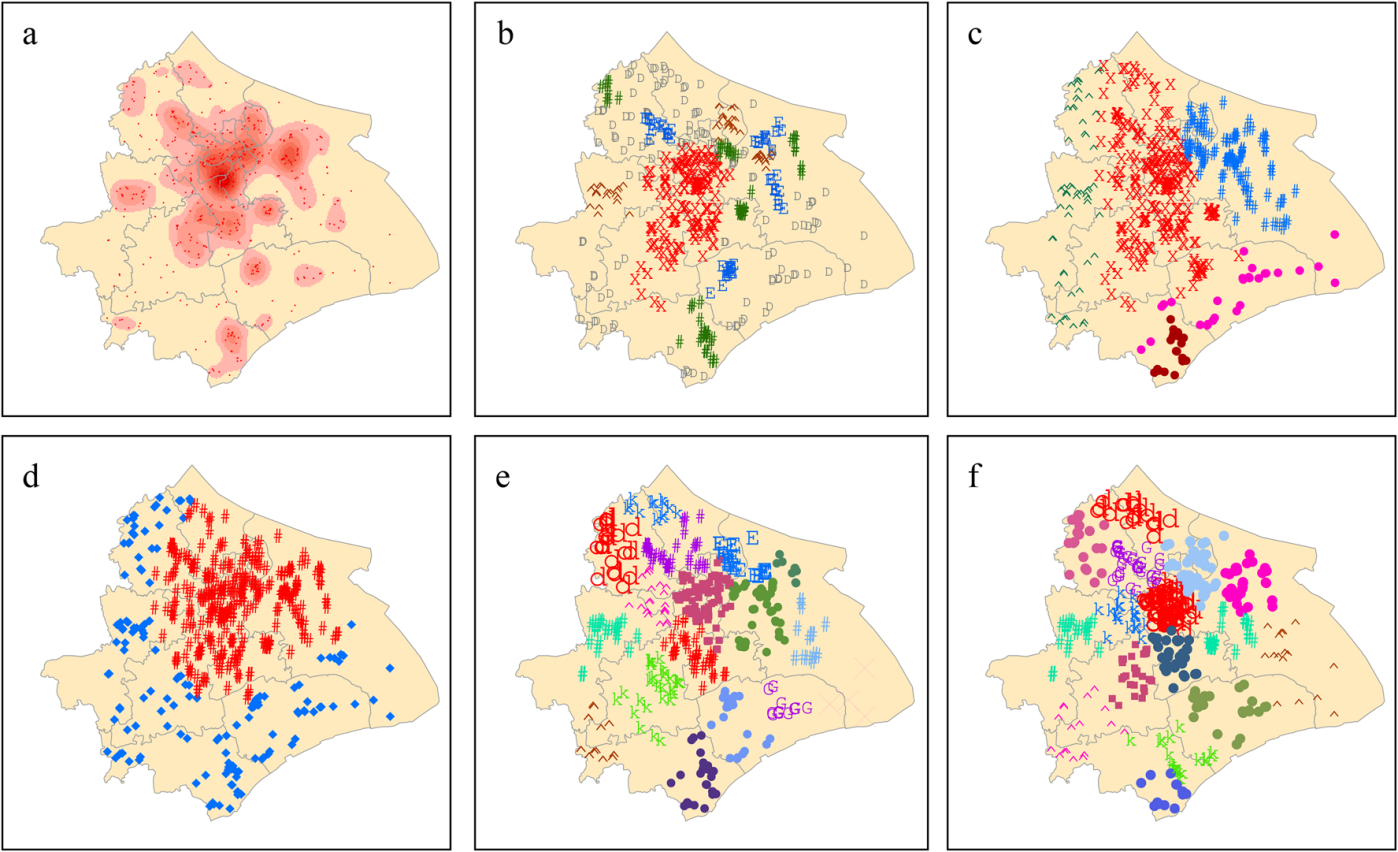
Identification of industrial clusters with different methods: (**a**) the result from the kernel function, (**b**) the result from EBC, (**c**) the result from GMM-EM, (**d**) the result from DBSCAN, (**e**) the result from complete-link, (**f**) the result from K-means.

Then we compare the results of different clustering methods with those of kernel density analysis to verify whether the clustering methods identify dense areas and noise points. The results of the optimal parameter from four clustering methods are shown in Fig. 4c–f. We perform kernel density analysis using square cells with sides of 400 meters. As shown in Fig. 4a, the results of kernel density analysis display vague boundaries of potential clusters. By comparing Fig. 4b–f with Fig. 4a, it is not difficult to see that the dense area identified by EBC is the most similar to Fig. 4a. In addition to the central area of Shanghai City, in Fig. 4b, the green area at the upper left, the brown area at the left, the green area at the bottom, and the blue area at the bottom show that EBC accurately identified regions with different local densities. In contrast, the dense regions and the regions of low object density (noise) can not be distinguished from Fig. 4c,e,f. In Fig. 4e, complete-link cannot merge the relatively compact and dense clusters in the central area, and split the large clusters in the center of the city, eroding the compactness of clusters. The results from GMM-EM, K-means and EBC have certain similarities, but GMM-EM and K-means can’t eliminate the noisy points. According to Fig. 4d, the red area is the dense area identified by the DBSCAN algorithm, and the blue area is the noise. However, compared with the kernel density analysis results in Fig. 4a, the clustering results of DBSCAN are not effective enough to identify the local dense regions with different densities. In general, compared with GMM-EM, complete-link and K-means, EBC distinguishes noise points between dense areas; compared with DBSCAN, EBC can identify local dense areas; compared with complete-link and K-means, EBC can ensure the continuity of the identified dense areas, without cutting the sufficiently dense areas. The results show that EBC is more effective than the four algorithms in identifying dense areas and noise points, and more suitable for analyzing industrial clusters.

EBC identifies a large cluster in the center of Shanghai City and several small clusters around it. Complete-link recognizes the relatively separated clusters in the surrounding area but it cannot merge the relatively compact and dense clusters in the central area, and split the large clusters in the center of the city, eroding the continuity. Meanwhile, DBSCAN has fallen into the trap of continuity and cannot effectively divide high-density clusters. The results from GMM-EM, K-means and EBC have certain similarities, but GMM-EM and K-means can’t eliminate the noisy points. Generally speaking, on firms’ location data set, EBC can identify clusters of various shapes and scales, and higher densities.

## Conclusion

We have proposed an ellipse-based approach to distinguish highly concentrated areas from noisy points, so as to analyze the industrial multi-agglomeration pattern and degree of industrial agglomeration in continuous space, with a high degree of consistency in cluster representation, quantification and identification. All data points are reordered into a number of sequences by using group-based nearest neighbors, and each group of points in the sequences, examined as a potential cluster, is represented by an ellipse in terms of elliptic parameters. Clustering is reformulated as the problem of identifying target ellipses among a number of potential ellipses, with significant changes (especially in the area) used as the cutoff criterion for determining clusters and the maximum sum of areas as the objective function.

We have illustrated the utility of EBC by applying it to an industrial data set. Without the necessity to know prior information, as a data-driven clustering method, EBC distinguishes high density areas from noise, with its performance under DBI obviously better than the other four methods. Besides, our ellipse-based approach reveals detailed information about industrial agglomeration, including the number, location, boundary, and degree of industrial clustering. In the case of Shanghai City, the agglomeration areas identified by our approach are highly consistent with the distribution of high-density areas identified in the kernel density analysis. The 339 listed manufacturing firms are distributed in 13 clusters, and the average density of agglomeration areas is 2.2 times that of the total density.

This study proposed an industrial agglomeration identification method using ellipses. Notably, EBC is a flexible and adjustable method due to the rich spatial information revealed by ellipses. In the initial process of ordering, the second-nearest neighbor is optional, so as to expand the collection of compactness sequences. The use of weighted standard deviation ellipse makes it possible to identify industrial agglomeration based on attribute data such as employment and industrial output.

In the future, more work is needed to improve our ellipse-based model. We can use elliptic parameters instead of distance metric. The distance metric can be replaced by elliptic parameters to determine group-based nearest neighbors. Besides, the appropriate use of shape and density constraints in the sorting process may improve the efficiency of the algorithm. In addition, on the basis of identifying the absolute agglomeration of industries, further immediate lines of research could consider the comparison of the agglomeration between different industries and regions, and explore whether the actual distribution of industrial agglomeration deviates from a random distribution.

## Supplementary Information

Supplementary Information 1.

Supplementary Information 2.

## Data Availability

The datasets used during the current study are included in the Supplementary information file.
