# Supplementary material for: A new method for identifying industrial clustering using the standard deviational ellipse
Source: Sci Rep. 2023 Jan 11;13:578. doi: 10.1038/s41598-023-27655-8 (PMC9834335; doi:10.1038/s41598-023-27655-8)
Supplement: Supplementary file 1 — Supplementary Information 1. [file 41598_2023_27655_MOESM1_ESM.pdf]

A new method for identifying industrial clustering using the standard deviational ellipse

Zi-Wei Zhao[1,2], Zuo-Quan Zhao[1,2], and Pei Zhang[3]

[1] School of Public Policy and Management, University of Chinese Academy of Sciences, 100049 Beijing, China

[2] Institutes of Science and Development, Chinese Academy of Sciences, 100190 Beijing, China

[3] Key Laboratory of Regional Sustainable Development Modeling, Institute of Geographic Sciences and Natural Resources Research, China Academy of Sciences, 100101 Beijing, China

Supplementary Note 1: symbols description

The symbols and notations used in this paper are listed as follows:

Symbols	Notations
E, D	Two disjoint sets
$GNN(D,E)$	The group-based nearest neighbor of D within E
$d(\cdot,\cdot)$, $d_{avel}(\cdot,\cdot)$	Functions of distance
X, x_i	The data set and sample points
A_{ij}	The compactness sequence of j points starting from x_i
B_{ij}	The complement of A_{ij} in x_i
Z	Spatial compactness matrix
z_{ij}	The group-based nearest neighbor of $A_{i,j-1}$ within $B_{i,j-1}$
$\hat{x_k}$, $\hat{y_k}$	The ordinate and horizontal coordinate of z_{ik} in A_{ij}
θ,σ_x, σ_y	The azimuth and the length of the half-axis of the ellipse corresponding to A_{ij}
M_{mc}, M_{area}, M_d, M_s, M_a	Elliptic parameter matrices
$Area$	Area gradient matrix
H_1, H_2, F	The sets of clusters
H	Clustering adjacency matrix

Supplementary Note 2: algorithms for important steps of EBC

Algorithm 1 Calculate Spatial Compactness Matrix

Input: the data set X, n
Output: the Spatial Compactness Matrix Z

k=1, Z = Null
for p in X **do**
  $Z_{p,k} = X_p$
  $A_{p,k} = Z_{p,k}$
end for
while k<n **do**
  for $pinX$ **do**
    $Z_{p,k+1} = GNN(A_{p,k}, X - A_{p,k})$
    $A_{p,k+1} = A_{p,k} \cup \{z_{i,j+1}\}$
  end for
  k=k+1;
end while
return Z =0

Algorithm 2 Determine candidate clusters

Input: the compactness sequences A, the density matrix M_d, the area gradient matrix $Area$, the size of X-n, xi
Output: the set of candidate clusters F

$J = \{j(i) \| m_3(i, j(i)) = max(m_3(i,t)), i, t = 1, 2, \cdots, n\}$
$F =, i = 1$
while $i < n$ **do**
  $k = j(i) + 1$
  while $Area_{p,k} < \xi$ **do**
    k=k+1;
  end while
  $F = F \cup A_{p,k}$
  $i = i + 1$
end while
return F =0
